# Inhibition of CX3CL1 by treadmill training prevents osteoclast-induced fibrocartilage complex resorption during TBI healing

**DOI:** 10.3389/fimmu.2023.1295163

**Published:** 2024-01-12

**Authors:** Xiao Liu, Mei Zhou, Jindong Tan, Lin Ma, Hong Tang, Gang He, Xu Tao, Lin Guo, Xia Kang, Kanglai Tang, Xuting Bian

**Affiliations:** ^1^ Department of Sports Medicine, Southwest Hospital, Army Medical University, Chongqing, China; ^2^ Pancreatic Injury and Repair Key Laboratory of Sichuan Province, The General Hospital of Western Theater Command, Chengdu, Sichuan, China

**Keywords:** tendon-bone insertion healing, treadmill training, osteoclast, CX3CL1, CX3CR1, bone eminences

## Abstract

**Introduction:**

The healing of tendon-bone injuries is very difficult, often resulting in poor biomechanical performance and unsatisfactory functional recovery. The tendon-bone insertion has a complex four distinct layers structure, and previous studies have often focused on promoting the regeneration of the fibrocartilage layer, neglecting the role of its bone end repair in tendon-bone healing. This study focuses on the role of treadmill training in promoting bone regeneration at the tendon-bone insertion and its related mechanisms.

**Methods:**

After establishing the tendon-bone insertion injury model, the effect of treadmill training on tendon-bone healing was verified by Micro CT and HE staining; then the effect of CX3CL1 on osteoclast differentiation was verified by TRAP staining and cell culture; and finally the functional recovery of the mice was verified by biomechanical testing and behavioral test.

**Results:**

Treadmill training suppresses the secretion of CX3CL1 and inhibits the differentiation of local osteoclasts after tendon-bone injury, ultimately reducing osteolysis and promoting tendon bone healing.

**Discussion:**

Our research has found the interaction between treadmill training and the CX3CL1-C3CR1 axis, providing a certain theoretical basis for rehabilitation training.

## 1 Introduction

The tendon-bone insertion is a critical site of attachment for tendons to bone, facilitating the transfer of force generated by muscle contractions. This complex structure encompasses the distal end of the tendon, the transitional area where the tendon inserts into the bone, and the mineralized bone eminences. Moreover, it is present in various regions of the body, such as the rotator cuff, cruciate ligament of the knee, and Achilles tendon. The incidence of rotator cuff tears, for instance, has been on the rise due to the popularity of sports (1–3). The high incidence of the disease is accompanied by harmful effects on people’s normal life and work activities. Including high surgical costs and various complications (4–10). Ultimately, even after successful surgery and postoperative rehabilitation, there is still the challenge of functional recovery that plagues physicians and patients (11–13). Therefore, promoting injury repair at the tendon-bone insertion remains a hot issue in the field of bone and joint research, and there are still many challenges to be addressed.

The fibrocartilage complex is a crucial component of the tendon-bone insertion, which features four distinct layers, including the tendon, noncalcified fibrocartilage layer, calcified fibrocartilage layer, and bone (14, 15). Under physiological conditions, the tensile modulus of tendon is approximately 200 MPa and that of bone is 20 GPa (16). The significantly different stiffness leads to stress concentration and failure at the interface between them. Functionally graded structures such as the fibrocartilage complex effectively reduce stress concentration by providing an ordered gradient of materials that efficiently transfer forces from the muscle to the bone via the tendon (17–20). Prior studies have focused primarily on the fibrocartilage layer, but recent research has demonstrated the importance of the bone eminences of the tendon-bone insertion in repair and regeneration.

Bone eminences arise from the gradient mineralization of the fibrocartilaginous complex and play a crucial role in providing a stable anchor point for tendon attachment. This structure interconnects with the tendon and facilitates stress dissipation, improving the biomechanical strength of the tendon-bone insertion (21). During the development of the tendon-bone insertion, a group of SOX9+/SCX+ progenitor cells at the tendon-bone interface, distinct from the limb bone, differentiate into chondrocytes after being regulated by cytokines such as TGF-β and BMP4, after which the chondrocytes gradually mineralize and transform into osteoblasts, eventually forming a bone eminence structure that interconnects with the tendon (22). The repair and regeneration of bone eminences is a critical step in the tendon-bone healing process, where osteoclasts resorb damaged bone and replace it with new bone formed by osteoblasts. In other tendon-bone insertions that do not require a fibrocartilage layer for transition, such as the anterior cruciate ligament, it has been proven that maintaining bone mass by inhibiting osteolysis induced by osteoclasts improved tendon-bone healing (23–26). Therefore, we believe that promoting the repair and regeneration of bone eminences after tendon-bone injury could potentially be an important breakthrough to solve the tendon-bone healing challenge.

Regeneration of bone eminences at the tendon-bone insertion is the process by which osteoclasts take up damaged bone and replace it with new bone formed by osteoblasts (27). Osteoclasts originate from hematopoietic stem cells and are formed by fusion stimulated by factors such as M-CSF and RANKL (28, 29), and mature osteoclasts degrade bone by synthesizing and secreting acids and proteolytic enzymes (30). Several studies have shown that inhibition of osteoclast differentiation and its activity can effectively inhibit bone resorption, thereby protecting bones from excessive destruction (31–35). Osteoclasts also play an important role in tendon-bone healing, and our previous studies have shown that treating tendon insertion injuries by using exosomes rich in miR-6924-5p can effectively inhibit the formation of osteoclasts, thereby inhibiting osteolysis and improving tendon healing strength (36). In addition, inhibition of osteoclastic activity with osteoprotegerin (OPG) significantly promotes an increase in bone mass at the tendon interface and improves stiffness at the tendon insertion (37).

Rehabilitation training, such as continuous passive motion (CPM) after rotator cuff tear surgery, has been shown to promote healing outcomes (38, 39), including reduced pain (40), accelerated functional recovery (41), and improved joint mobility (42, 43). However, the effect of mechanical stimulation on tendon-bone insertion during training only partially explains the beneficial effects of rehabilitation training. Running training, for instance, not only plays a role in promoting muscle contraction (44, 45) but also regulates the synthesis and secretion of various cytokines involved in lipid metabolism (46), insulin resistance (47), inflammation (48), learning and memory (49), as well as hormones and nerve growth factors (50–56). The effect of running training on the synthesis and secretion of cytokines in the body has been repeatedly demonstrated, but its impact on the process of tendon-bone healing remains understudied.

Therefore, starting from clinical rehabilitation training, this study will focus on the effect of treadmill training on the repair of the bone eminences of the tendon-bone insertion, and delves into the changes, effects, and mechanisms of local cytokines during this process, providing new directions and ideas for the study of tendon-bone insertion healing.

## 2 Materials and methods

### 2.1 Animal model and study design

The male C57BL/6 mice used in this study were 8 weeks old and weighed approximately 23 g. These mice were subjected to standard procedures and guidelines for animal care and use, as approved by the Animal Ethics Committee (AMUWEC20210782). For Micro CT detection, we selected the ipsilateral lower limbs (n=6) of six mice without surgery as the normal group and compared them with the control group (only surgery) and the experimental group (postoperative treadmill training). The mice undergoing surgery were subjected to the following treatments: regular treadmill training (training group) according to the experimental protocol, free movement within the cage after surgery (control group), and local injection of antagonist AZD8797 (AZD8797 group) (MCE HY-13848) after surgery.

For animal models, according to our previously published method (57), tendon insertion injury and repair were performed on mice. As per the approved animal care and use protocol use 0.1 mg/kg BW of Buprenorphine SC 2 hour prior to skin incision (58, 59). The mice were subjected to intraperitoneal injection of 0.5% sodium pentobarbital (dose: 10 ml/kg body weight) for anesthesia purposes (60), followed by sterilization of the hind limbs and a 4-mm-long incision made along the long axis of the tibia at the right side of the insertion of the tibia and the heel bone. An insulin needle was used to open the bone tunnel from right to left 1 mm from the end of the heel bone, and a 6-0 polydioxanone (PDS) suture (Ethicon) was passed through the bone tunnel with a needle from the right side, through the tendon from the lower left approximately 1 mm from the end of the Achilles tendon, and again through the tendon from above to the lower right, preserving the end of the thread. The Achilles tendon was then cut at the end of the tendon immediately adjacent to the heel bone, preserving the connective tissue around the tendon to prevent retraction, and the fibrocartilage layer at the tendon-bone insertion was carefully scraped away using a surgical blade until the cancellous bone was exposed. Finally, the skin was sutured with interrupted sutures and disinfected. Starting from 4 hours after surgery, administer buprenorphine analgesia every 6-8 hours for 3 consecutive days (0.1mg/kg SC) (58, 59). Then mice were allowed to move freely in the cage until the initiation of the running stage of the training phase.

### 2.2 Treadmill training

The training program for the treadmill training group began on the seventh day after surgery and entailed a 30-minute daily regimen. The exercise intensity was gradually increased, starting at 5 m/min on the seventh day and progressing to 7 m/min on the eighth day, 9 m/min on the ninth day, and 10 m/min from the tenth day onward. The voltage was set to 1.0 mA, and a maximum of 5 mice were allowed to run on each runway. The training session was programmed to end automatically after 30 min, and the mice were promptly returned to their respective cages to move freely. All mice in the surgical group were euthanized at 14 days postoperatively, and samples of tendon-bone insertion tissue were collected for subsequent analysis.

### 2.3 Cytokine microarray analysis

The cytokine microarray analysis was entirely completed by ‘Wayne Biotechnology (Shanghai), Inc’. The specific steps are as follows. Firstly, the sample protein was extracted using chip specific lysate and subjected to BCA protein quantification. Then repeat the dialysis of the sample twice using a dialysis tube and continue overnight. Next, follow the hybridization standard process and reagent kit provided by Raybiotech company for chip testing. After sealing the chip containing the sample at room temperature for 30 minutes, wash the chip 5 times with 1x Wash Buffer I, each time for 5 minutes. Next, add 400 μL 1x Cy3 Conjugated Streptavidin to each well, apply adhesive strips, place in a horizontal shaker at 70 rpm, room temperature, and incubate for 2 hours. Finally, wash the chip three times using 1x Wash Buffer I, place the chip in a small chip centrifuge at 1000 rpm, centrifuge for 3 minutes to dry, and use an Agilent SureScan Dx Microarray Scanner chip scanner to scan the chip at 532 nm, Power (100%) conditions.

### 2.4 Histological evaluation

Tissue samples were harvested from the tendon-bone insertion of mice at 14 days post-surgery and were fixed in a paraformaldehyde solution of more than 4% for 48 h at 4°C. Next, the samples were subjected to two washes and decalcified for 72 h at 4°C by EDTA decalcification solution (Solarbio E1171). The samples were then washed again and incubated in a sucrose-paraformaldehyde solution (LEAGENE Cat : DF0144) for 48 h at 4°C. Next, the fixed tissue samples were treated by gradient dehydration and xylene transparency. Finally, the tissues were embedded in paraffin wax and sectioned at a thickness of 7 µm using a paraffin slicer.

Before performing the staining operation, the slices are sequentially subjected to baking, dewaxing, and gradient hydration steps for later use. Prepared sections were subjected to HE staining (Solarbio G1121) and TRAP staining (WAKO 294-67001) according to the instructions. The stained sections were then evaluated under a light microscope. One blinded researcher independently assessed and recorded the staining results. The tendon-bone insertion areas were observed and recorded at ×10 and ×40 magnification, and the results were recorded. The maturity of the tendon-bone insertion was scored based on the number of fibrocartilage cells, fibrocartilage cell alignment, collagen fiber continuity, collagen fiber orientation, Tidemark, cellularity, vascularity, inflammation, and eight other categories using HE staining (**Supplementary Table S5**). Each category was scored on a scale of 0-3, with a maximum total score of 24 indicating the best maturity of tendon-bone insertion healing. The number of osteoclasts in the tendon insertion was evaluated using TRAP staining, and the index was the number of osteoclasts at ×20 magnification. Two independent scorers, blinded to the groups, scored all specimens using a randomized method to minimize subjectivity. Any significant differences were re-evaluated and discussed.

### 2.5 Quantitative reverse transcriptase polymerase chain reaction

The collected tissue samples were rapidly frozen in liquid nitrogen and then pulverized under liquid nitrogen cooling on an ultraclean table, followed by the addition of 1 ml TRIzol to each 1.5 ml centrifuge tube containing the pulverized tissues. The lysed tissues were then fully mixed, and 200 μL of trichloromethane was added to each tube, which was then shaken vigorously for 30 s and allowed to stand for 3 min. The tubes were then centrifuged at 4 °C and 12400 rpm for 25 min, after which 400 μL of supernatant was aspirated and mixed with 400 μL of isopropanol. The mixture was inverted and allowed to stand for 10 min, followed by centrifugation under the same conditions for 15 min. The supernatant was discarded, and the precipitate was washed with 1 mL of 75% alcohol and centrifuged again for 5 min. This washing step was repeated twice, and then the alcohol was gently aspirated and discarded. The extracted RNA was dissolved in 50 μL of DEPC water, and the RNA concentration was measured under a spectrophotometer. Reverse transcription was carried out with a final mass of 1000 μg, followed by dilution of the reverse transcribed samples with 180 μL of DEPC water. A 10 μL PCR reaction system containing 2.5 μL cDNA, 2.5 μL primers, and 5 μL iTaq™ Universal SYBR Green SuperMix per well was then configured for fluorescence PCR. The PCR program included incubation at 95°C for 5 min, followed by 30 three-step temperature analyses. The ΔCt method was used for data analysis with GAPDH serving as the housekeeping gene.

### 2.6 Cell culture

Male C57BL/6 mice aged 8-12 weeks and weighing approximately 25 g were selected, sacrificed, and tissue samples were sterilized by immersion in 75% alcohol for 5 min. Primary monocyte-macrophages were extracted from the bone marrow cavities of the femur and tibia of the mice using the flushing method. The cells were harvested in suspension after 12 hours of culture at 30°C and 5% CO_2_, counted, and plated in α-MEM medium containing 10% FBS, 1% penicillin−streptomycin solution, and 50 ng/mL Recombinant Murine M-CSF (PeproTech 315-02) at a density of 2 million cells/well for 6-well plates and 40,000 cells/well for 96-well plates. The culture medium containing M-CSF was supplemented once on the second day of culture, and then the solution was changed on the fourth day. At the same time, Recombinant Murine sRANK Ligand (100ng/ml) (PeproTech 315-02) was added additionally. At the beginning of RANKL induction, intervention factors were added, including: control group (DMSO), differentiation induction group (RANKL), CX3CL1 group (RANKL, recombinant CX3CL1 protein) (MCE HY-P72686), AZD8797 group (RANKL, recombinant CX3CL1 protein,AZD8797).The cell culture medium containing M-CSF, RANKL and intervention related cytokines was continuously replaced every day, and samples were collected on the fourth day and the seventh day of RANKL induction for PCR experiments and TRAP staining.

### 2.7 Immunofluorescence

After the paraffin sections of the tissue samples were dewaxed and hydrated, the sections were washed twice with PBS for 5 min each time. Subsequently, the sections were treated with 0.3% Triton and incubated at 37°C for 30 min. After washing twice with PBS, the sections were blocked with 3% BSA solution and incubated at 37°C for 30 min, followed by another wash with PBS. Mouse CTSK antibody (Invitrogen Cat# PA5-14270) was then applied to the sections at a dilution of 1:100 and incubated overnight at room temperature. After three washes with PBS for 10 min each, AF588-coupled donkey anti-rabbit secondary antibody (Invitrogen Cat# PA5-14270) was added to the sections at a dilution of 1:200, followed by another wash. Nuclear restaining was performed using a DAPI blocker, and the sections were sealed, left to dry, and observed under a fluorescence microscope. CTSK-positive cells at the tendon-bone insertion were counted at 20x magnification. Scoring was performed according to previously established protocols. All procedures were performed in triplicate, and the data were obtained from at least three independent experiments.

### 2.8 Bone morphometric analysis

Following the euthanization of the mice, samples from the mid-gastrocnemius to the foot were collected and fixed in paraformaldehyde for 48 h and then transferred to PBS. The samples were subjected to micro computed tomography (CT) scanning using an energy of 60 kVp, an intensity of 166 μA, an aluminum 0.25 mm filter, and a resolution of 7 μm. The region of interest was defined as 5 mm from the tendon-bone insertion. Parameters such as bone mineral density (BMD), trabecular thickness (Tb, Th, mm), trabecular spacing (mm), tissue mineral density (TMD), bone volume (BV), and bone volume fraction (BV/total volume) were measured.

### 2.9 Biomechanical testing

Upon rapid removal from the mid gastrocnemius to the foot post-execution, the tendon insertion was immediately subjected to biomechanical strength testing while periodically being sprayed with PBS to maintain its moisture content. The tissue ends were securely fixed to the fixture, with caution exercised to avoid attaching the tendon-bone insertion to prevent any potential impact on experimental outcomes. Subsequently, the tissue was elongated at a constant rate of 0.1 mm/sec without any pre-tensioning until the sample was fractured. During the testing process, tensile strength, stiffness and elongation data were continuously recorded and automatically analyzed by the testing system to generate load-stress curves and stress−strain curves.

### 2.10 Behavioral test and CatWalk gait analysis

Open field behavioral testing and assessment were carried out following established protocols from previous studies conducted by our research group (61). The apparatus for the test was constructed using gray plywood and measured 40 × 40 cm, with walls 30 cm high. Each mouse was placed in the center of the open field, and its movements were recorded for 10 min using a camera positioned above the apparatus. EthoVision 11.0 (Noldus) was used for further analysis of the recorded data. To allow for acclimation, each mouse was placed in the apparatus for 10 min prior to the actual testing. Following the test, the apparatus was thoroughly cleaned using 70% ethanol and a clean paper towel. The distance traveled (cm) and speed (cm/s) were measured and recorded to reflect the basic locomotor ability of the mice. The CatWalk XT Gait Analysis System (Noldus) was used to automatically record movement and analyze the motor function of rats. Before testing, each mouse become familiar with the environment, then they were placed on the walkway and allowed to move freely. A high-speed camera was used to record the mouse’s paw prints, and software was used to automatically analyze the mouse’s LH stride, RH stride, and average speed.

### 2.11 Statistical analysis

Statistical analyses were performed with GraphPad Prisma 8.0 software, and data are shown as the mean ± standard error. Perform one-way analysis of variance on the data. The differences were considered significant at p < 0.05.

## 3 Results

### 3.1 Treadmill training improves tendon-bone healing by inhibiting osteolysis

**Figure 1:**
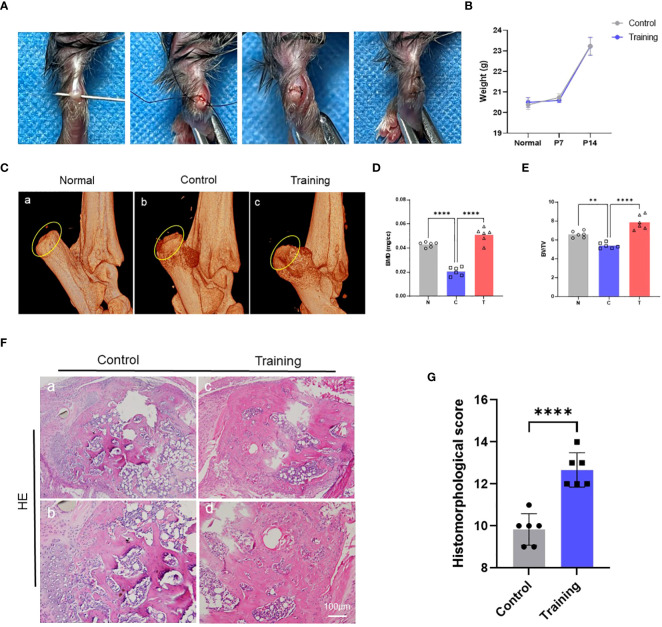
Treadmill training promotes tendon-bone healing by inhibiting osteolysis: **(A)** Construction procedure of the tendon-bone insertion injury healing model; **(B)** The weight changes in mice after surgery were recorded; **(C)** The morphology of the bone bulge at the tendon-bone insertion was observed by micro-CT and statistically analyzed for BMD **(D)** and BV/TV **(E)**; **(F)** HE staining of the tendon-bone insertion tissue and histological scoring **(G)**; N: normal, C: control, T: training. Data are shown as the mean ± SEM and n = 6 per group per experiment. Statistical significance was determined using one-way ANOVA with Dunnett’s multiple-comparisons test with **P < 0.05 and ****P < 0.01.

To examine the effect of treadmill training on tendon-bone healing, we created an animal model of tendon-bone injury healing (**Figure 1A**) and immobilized the hind limbs of mice in a functional position immediately after surgery to restrict movement and minimize the inflammatory response in the early postoperative period. Treadmill training began on day 7 after surgery, and the training protocol was based on our previous study. We monitored the body weight of the mice before surgery, 7 days after surgery, and 28 days after surgery and found that treadmill training did not have a significant effect on the body weight of the mice (**Figure 1B**). Micro-CT was performed on the tendon-bone insertion of the hind limbs of mice 14 days after surgery (i.e., 7 days of treadmill training), and 3D reconstructions showed intact bone augmentation at the tendon-bone insertion in the normal group. In contrast, bone augmentation lysis was evident in the control group, and osteolysis was suppressed in the treadmill training group (**Figure 1C**). We selected the bone augmentation part of the fibrocartilage complex for BMD and BV/TV analysis, which revealed that the BMD and BV/TV of the control group were significantly lower than those of the normal group, whereas the BMD and BV/TV of the treadmill training group were significantly higher than those of the control group (**Figure 1D, E**, **Supplementary Table S1**). Our results indicate that osteolysis occurs in the bone augmentation part of the fibrocartilage complex during the healing process of tendon insertion injury, and treadmill training can significantly inhibit osteolysis, promoting the repair of the bone eminences of the tendon insertion fibrocartilage complex.

### 3.2 Treadmill training inhibits osteoclast formation at the tendon-bone insertion

**Figure 2:**
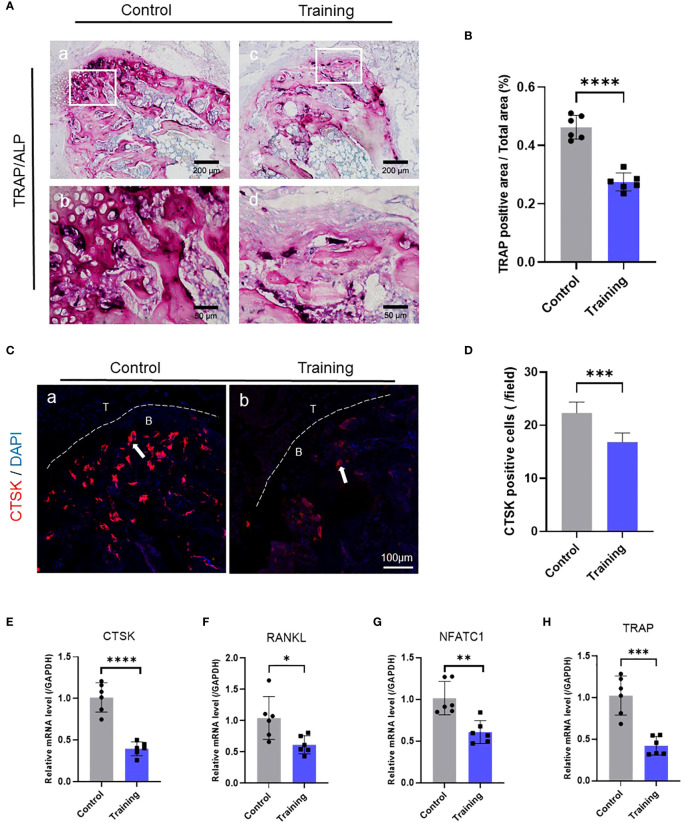
Treadmill training inhibits osteoclast formation: **(A)** TRAP staining of the tendon-bone insertion and counting the number of positive cells **(B)**; **(C)** Immunofluorescence staining using CTSK antibody and counting the number of positive cells near the tendon-bone interface **(D)**; **(E–H)**. PCR analysis of the gene expression levels of CTSK, RANKL, NFATc1 and TRAP at the tendon-bone insertion. Data are shown as the mean ± SEM and n = 6 per group per experiment. Statistical significance was determined using one-way ANOVA with Dunnett’s multiple-comparisons test with * P < 0.05, ** P < 0.01, *** P < 0.001, and **** P < 0.0001.

To further explore how treadmill training inhibited osteolysis in the osseous bulge of the tendon-bone insertion, we conducted histochemical staining of the tendon-bone insertion in mice 14 days after surgery. Moreover, HE staining was used to assess the gross histological manifestations of the tendon-bone insertion and was analyzed with reference to the histological scoring scale of our previous study, which revealed that the treadmill training group had significantly higher histological scores than the control group (**Figure 1F, G**, **Supplementary Table S2**). We also examined osteoclasts at the tendon-bone insertion in mice using TRAP staining and found that the number of osteoclasts in the treadmill training group was significantly lower than that in the control group (**Figure 2A, B**). Additionally, CTSK immunofluorescence staining and positive cell counts of the tendon-bone insertion tissue showed that the number of CTSK-positive cells in the treadmill training group was significantly lower than that in the control group (**Figure 2C, D**). We performed polymerase chain reaction to measure the expression of osteoclast-related genes (**Supplementary Table S3**), and the results showed that the expression of TRAP, NFATc1, CTSK, and RANKL was significantly inhibited in the treadmill training group compared to the control group (**Figure 2E-H**). These findings suggest that during the repair process of tendon-bone insertion injury in mice, a large number of differentiated mature osteoclasts were present in the bone augmentation part of the fibrocartilage complex, and treadmill training significantly inhibited osteoclast formation and aggregation. This indicates that treadmill training can reduce osteolysis in the bone augmentation portion of the fibrocartilage complex by inhibiting osteoclast formation.

### 3.3 Treadmill training significantly inhibits CX3CL1 secretion at the tendon-bone insertion

**Figure 3:**
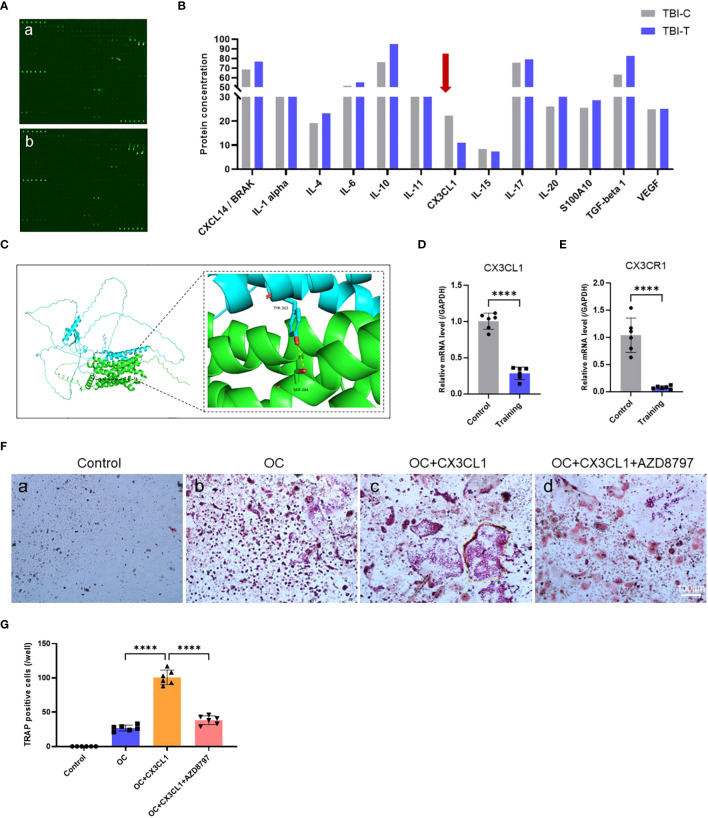
Treadmill training inhibits osteoclast formation by suppressing CX3CL1 secretion: **(A, B)** Cytokine microarray analysis technique to detect cytokine secretion levels in the tendon-bone insertion; **(C)** The ZDOCK score values and best pose interaction of CX3CL1 and CX3CR1. **(D, E)**. PCR analysis of gene expression levels of CX3CL1 and CX3CR1 in the tendon insertion. **(F, G)** Osteoclast induction of primary monocyte-macrophages with the addition of CX3CL1 recombinant protein or CX3CR1 antagonist protein (AZD8797) and TRAP staining of culture results and counting of positive cells. Data are shown as the mean ± SEM and n = 6 per group per experiment. Statistical significance was determined using one-way ANOVA with Dunnett’s multiple comparisons test with ****P < 0.0001.

To further investigate the mechanism by which treadmill training inhibits osteoclast formation, we conducted cytokine microarray analysis on tendon-bone insertion tissues. Based on the raw data provided by Cytokine microarray analysis, a total of 308 cytokines were detected in this study. Based on the “ Comparison Results between Groups”, we screened “Ratio-Ms TBI Tvs-Ms TBI C” under the condition of “≥ 2 or ≤ 0.5”, and ultimately selected 17 cytokines with significant multiple differences (**Supplementary Table S6**). We reviewed the tissue distribution and function of 17 cytokines selected from the material methods on NCBI and GeneCards, as well as the relevant literature that has been published. Finally, we chose CX3CL1(Fractalkine). Our results showed that treadmill training significantly inhibited the secretion of CX3CL1 compared to the control group, while no significant differences were found for IL-1α, IL-10, TGF-β, and IL-4 (**Figure 3A, B**). Since CX3CR1 is a CX3CL1-specific receptor expressed in osteoblast progenitor cells, we calculated their ZDOCK score values and their best pose interaction, which revealed that CX3CL1 and CX3CR1 formed a connection with amino acid sites such as TYR362-SER284 (**Figure 3C**). The relevant PCR results also confirmed the involvement of CX3CR1 (**Figure 3D, E**). These findings suggest that the CX3CL1-CX3CR1 axis may be one of the key proteins that links osteoblasts to osteoclasts and mediates the effect of treadmill training on inhibiting osteoclast formation.

### 3.4 The CX3CL1-CX3CR1 axis regulates osteoclast formation

**Figure 4:**
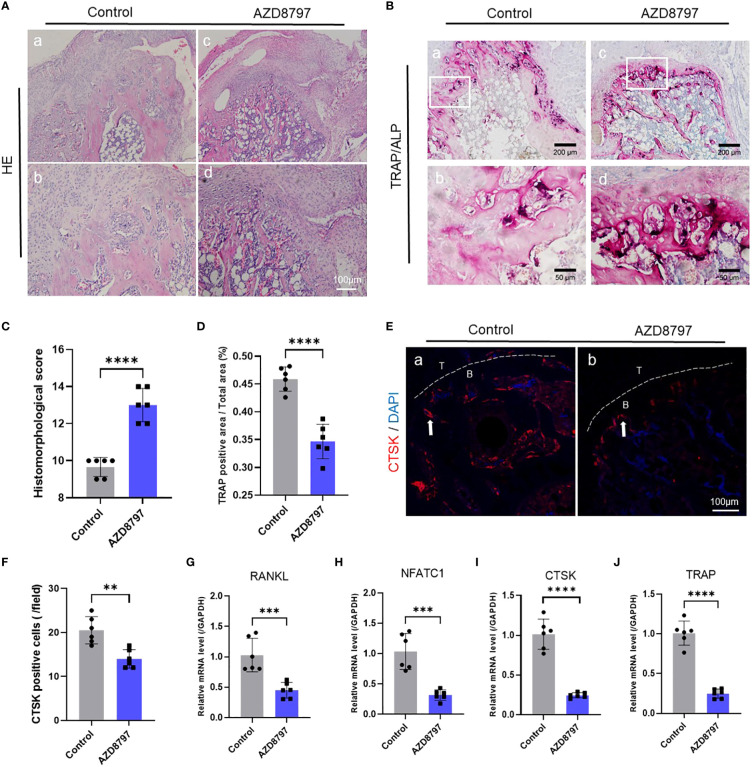
Antagonism of CX3CR1 inhibits osteoclast formation at the tendon-bone insertion: **(A)** The staining and histological scoring of the tendon-bone insertion tissue **(C)**;**(B)** TRAP staining of the tendon-bone insertion and counting the number of positive cells **(D)**; **(E)** Immunofluorescence staining of the tendon-bone insertion using CTSK antibody and counting the number of positive cells at the tendon-bone interface **(F)**. **(G-J)**. PCR was performed to measure the gene expression levels of CTSK, RANKL, NFATc1 and TRAP at the tendon-bone insertion site. Data are shown as the mean ± SEM and n = 6 per group per experiment. Statistical significance was determined using one-way ANOVA with Dunnett’s multiple-comparisons test with **P < 0.01, ***P < 0.001 and ****P < 0.0001.

To investigate the role of the CX3CL1-CX3CR1 axis in osteoclast formation, we conducted both *in vitro* and *in vivo* experiments. *In vitro* experiments, we identified a protocol for inducing osteoclast differentiation through literature review (62–64). First, we extracted mouse bone marrow-derived monocytes and induced mature macrophages after 4 days of culture under M-CSF, followed by osteoclast induction using recombinant CX3CL1 protein and CX3CR1 antagonist protein AZD8797. We then performed TRAP staining and observed cell morphology and positive cell counts after 7 days of induction. The results showed that recombinant CX3CL1 protein significantly promoted osteoblast formation and possessed a more mature morphology, but this promotion was significantly abolished by the simultaneous addition of AZD8797 (**Figure 3F, G**). In addition, *in vivo* experiments were conducted in a mouse model of tendon-bone insertion injury healing, where AZD8797 was injected on postoperative day 14. Tissue samples from the tendon-bone insertion were analyzed histologically, and the results showed that the histological score of the tendon-bone insertion was significantly higher than that of the control group after *in vivo* injection of AZD8797 (**Figure 4A, C**; **Supplementary Table S4**). Furthermore, TRAP staining (**Figure 4B, D**) and CTSK immunofluorescence staining results (**Figure 4E, F**) revealed that the number of osteoclasts was significantly lower after the use of AZD8797 than in the control group. Finally, PCR detection of osteoclast-associated gene expression showed that the expression of TRAP, NFATc1, CTSK and RANKL was significantly inhibited after the use of AZD8797 (**Figure 4G-J**). These results provide evidence that the CX3CL1-CX3CR1 axis plays a significant role in regulating the osteoclast formation process.

### 3.5 Antagonism of CX3CR1 promotes functional recovery after healing of tendon-bone insertion injury

**Figure 5:**
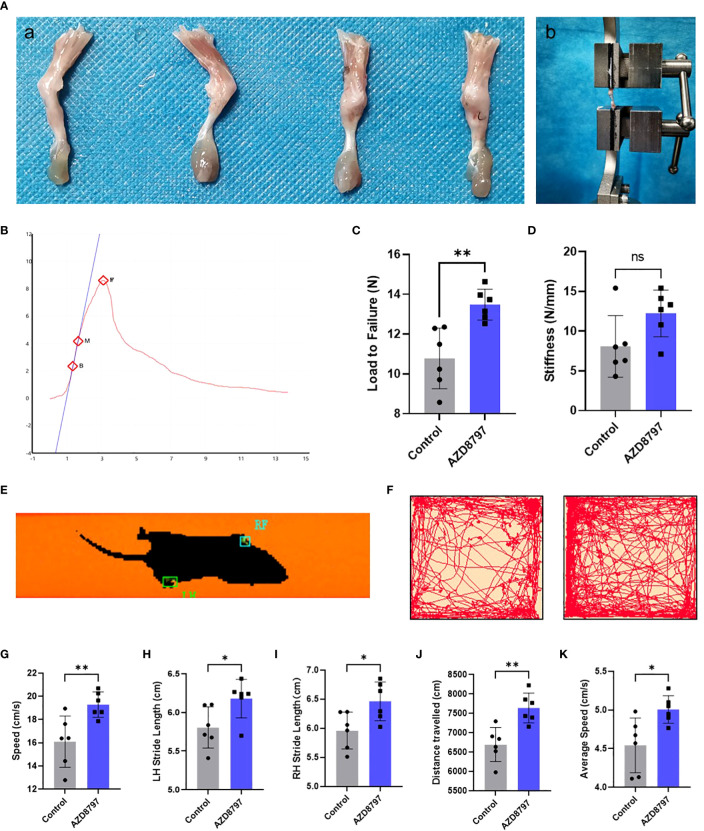
Antagonizing CX3CR1 improves biomechanical strength and promotes functional recovery of the tendon-bone insertion: **(A)** Morphology of the tendon-bone insertion 28 days after surgery; **(B)** Schematic diagram of biomechanical strength testing; **(C, D)**. Time−load curves and statistics of failure load and stiffness for biomechanical strength testing of the tendon-bone insertion; **(E-K)**. CatWalk gait analysis and open-field experiments with statistics of path, velocity and distance traveled by mice. Data are shown as the mean ± SEM and n = 6 per group per experiment. Statistical significance was determined using one-way ANOVA with Dunnett’s multiple-comparisons test with *P < 0.05, **P < 0.01 and ns, no significance.

To confirm the effect of the CX3CL1-CX3CR1 axis on tendon-bone healing, we administered AZD8797 into the mouse model of tendon-bone insertion injury healing and performed functional tests at 28 days postoperatively. As seen in the figure, the healing of the tendon insertion was essentially complete at this time point (**Figure 5A**). We extracted the intact tendon insertion for biomechanical testing and measured the failure load and stiffness during pulling. The results showed that the healed tendon insertion had significantly higher failure load and stiffness in mice treated with AZD8797 than in the control group (**Figures 5B-D**). Additionally, we performed an open field experiment and CatWalk gait analysis to assess the mobility of the mice by recording their speed, LH stride length, RH stride length, distance traveled, and average speed during the experiment. The results showed that the mice treated with AZD8797 exhibited significantly higher mobility after healing than the control group (**Figures 5E-K**). In conclusion, our study provides evidence that the CX3CL1-CX3CR1 axis is involved in the regulation of tendon insertion healing after injury, and its inhibition can significantly enhance the biomechanical properties and locomotor ability of mice after healing.

## 4 Discussion

This study investigates the underlying mechanisms of treadmill training as a rehabilitation modality to promote the repair and regeneration of tendon-bone insertion in mice following injury. The research results emphasize the impact of the changes in local cytokine levels on tendon-bone insertion and the repair of the bone eminences of tendon-bone insertion. To achieve the study’s objectives, our research team designed a suitable intensity of treadmill training for mice, drawing from the experimental design and results presented by Lu et al. (65) and Wada et al. (66) and the published articles of our group. The outcomes of the study reveal that treadmill training effectively improves the healing of the tendon-bone insertion by enhancing its morphological and biomechanical recovery and promoting bone formation at the tendon-bone insertion. The study found that treadmill training exerts an inhibitory effect on osteoclast differentiation during tendon-bone healing, which might be a crucial factor contributing to the success of treadmill training in promoting tendon-bone insertion healing. While previous research has established a close relationship between treadmill training and osteoclast formation in improving osteoporosis (67–69), fewer reports exist on the effect of treadmill training on osteoclasts in the field of tendon-bone insertion healing. Thus, this study’s results contribute significantly to the literature by providing evidence for the potential of treadmill training as a rehabilitation modality for tendon-bone insertion healing.

Numerous reports have explored the impact of treadmill training on tendon-bone insertion. However, the majority of these studies treat treadmill training solely as a mechanical stimulus and neglect to consider the local changes in tendon-bone insertion. As a rehabilitative exercise, treadmill training has been shown to impact multiple systems of the body, as evident in previous studies. For example, Chen et al. (70) reported that treadmill training protects proliferative SCs from failure through the Igfbp7-Akt-mTOR axis, which could be an effective tool for muscle regeneration therapy. Other studies have shown that treadmill training can reduce α-synuclein proliferation in the brain and protect nigrostriatal dopaminergic neurons in mice, preventing neurodegenerative diseases such as Parkinson’s disease (PD), LB dementia (LBD), and multiple-system atrophy (MSA) (71). Additionally, reports have indicated that treadmill training may improve atherosclerosis by upregulating serum β-hydroxybutyric acid levels (72). In this study, we utilized cytokine microarray analysis technology to analyze mouse tendon-bone insertion tissues and discovered that treadmill training significantly altered the levels of tendon-bone insertion cytokines, particularly CX3CL1.

CX3CL1, also known as Fractalkine, is a unique member of the chemokine family in the form of a membrane-bound glycoprotein. Its sole specific receptor, CX3CR1, is expressed in a wide range of immune cells, including monocytes, macrophages, and T-cells, which are the reasons why CX3CL1, together with CX3CR1, plays a central role in a variety of inflammatory diseases (73). In acute nephritis, CX3CL1 expression is capable of independently recruiting a subpopulation of monocytes expressing the CX3CR1 receptor to the appropriate site, causing kidney injury (74). There is evidence that the interaction of CX3CL1 with its specific receptor CX3CR1 is involved in the development of non-alcoholic steatohepatitis and in the process of skin wound healing through chemotactic recruitment of macrophages and regulation of their migration and polarization (75, 76). In addition, CX3CL1 is involved in the pathology of atherosclerotic disease by promoting macrophage adhesion and inhibiting their release of tissue factor and tumor necrosis factor-a (TNF-a) (77).

In bone tissue-related areas, CX3CL1 plays an equally important role. Previous studies have demonstrated that the CX3CL1/CX3CR1 axis is involved in cartilage destruction due to osteoarthritis by inducing MMP-3 secretion from synovial fibroblasts through c-Raf, MEK, ERK, and NF-κB pathways (78). In addition, CX3CL1 has been recognized as one of the important immunological markers for the evaluation of osteoporosis risk and prognosis (79). The CX3CL1/CX3CR1 axis also plays an important role during bone remodeling. It has been shown that CX3CL1 is expressed in osteoblasts, whereas its receptor CX3CR1 is present in osteoclast progenitors (80). For the process of osteoblast differentiation, deletion of the CX3CL1/CX3CR1 axis leads to the dysfunctional expression of osteogenic transcription factors and bone matrix proteins by inhibiting the secretion of chemokines such as CCR1, affecting the functional maturation of osteoblasts at the early stage of their differentiation, which ultimately manifests as impaired mineral deposition (81). It has also been shown that CX3CL1 expression is consistently elevated during osteogenic induced differentiation of bone marrow mesenchymal stem cells, and may be involved in cell proliferation and osteogenic differentiation of bone marrow mesenchymal stem cells by regulating cell distribution and aggregation through the RUNX2 pathway, and by facilitating cellular mutual induction and paracrine secretion (82). In addition to this, the CX3CL1/CX3CR1 axis has an important role in osteoclast differentiation and functioning. Several studies have shown that during osteoarthritis and cartilage degeneration, a significant elevation of CX3CL1 promotes the chemotactic recruitment of osteoclast precursor cells to the site of injury, whereas its chemotactic activity can be significantly inhibited by the use of CX3CL1-neutralizing antibodies (83–85). During osteoclast differentiation, CX3CL1 was able to modulate macrophage polarization through the NF-κB pathway and promote RANKL-induced osteoclast formation (86–88), an effect that disappeared after blocking the binding of CX3CL1 to CX3CR1. The same result occurred in experiments in which osteoblasts were co-cultured with osteoclast precursor cells, and osteoclast differentiation was markedly inhibited after CX3CL1 was blocked using neutralizing antibodies (89).

To investigate the role of CX3CL1 in osteoclast differentiation, osteoclast progenitors were cultured with recombinant CX3CL1 protein and an antagonist protein of its receptor, CX3CR1. As expected, CX3CL1 significantly promoted the differentiation of primary monocytes to osteoclasts, while this effect was inhibited in the presence of its receptor antagonist. These findings are consistent with those of Muraoka et al. (88), who reported that CX3CL1 stimulates the differentiation of peripheral blood-derived monocytes with dendritic cells toward osteoclasts. However, Koizumi et al. (89) and Matsuura et al. (90) found that CX3CL1 promotes osteoclast migration, adhesion, and resorption but does not directly promote osteoclast differentiation and requires simultaneous involvement of osteoblasts to promote osteoclast differentiation (89, 90). Our results are not consistent with these findings, and we speculate that this discrepancy may be due to differences in culture conditions and protocols. CX3CL1 may need to be fully induced into osteoclast progenitor cells by M-CSF in monocytes before it can exert its effect, but we did not verify this hypothesis. Notably, we observed that antagonism of the CX3CL1 receptor significantly inhibited osteoclast formation at the tendon-bone insertion while achieving a similar improvement in tendon-bone insertion healing as treadmill training. This simultaneous validation provides compelling support for our hypothesis.

This study has some limitations that need to be addressed. First, although our experiment demonstrates the role of CX3CL1 in promoting tendon-bone insertion healing through treadmill training, we could not entirely verify its importance, as CX3CL1 was not entirely knocked out using a mouse genetics or virus. Second, we only verified the effect of bench training on osteoclast formation and did not explore other osteoclast functions, such as migration, autophagy, and bone resorption. Last, as bone formation is a result of the joint action of osteoblasts and osteoclasts, our experiments only focused on osteoclasts and not osteoblasts. To address these limitations, our future experimental plans include using more techniques to verify the importance of CX3CL1, improving our understanding of osteoclast function, and investigating the effect of treadmill training on osteoblasts and their communication with osteoclasts. As CX3CL1 is expressed in osteoblasts and acts on osteoclast progenitor cells, it is likely that treadmill training affects both cell types, and the CX3CL1-CX3CR1 axis may be a critical protein linking them.

In conclusion, our study demonstrates that treadmill training promotes the regeneration of the bone eminences of the tendon-bone insertion by inhibiting CX3CL1 secretion *in vivo*, which further inhibits osteoclast formation, ultimately promoting tendon-bone insertion healing in mice.

## Data availability statement

The original contributions presented in the study are included in the article/**Supplementary Material**. Further inquiries can be directed to the corresponding authors.

## Ethics statement

The animal study was approved by Animal Ethics Committee of the Army Medical University. The study was conducted in accordance with the local legislation and institutional requirements.

## Author contributions

XL: Investigation, Methodology, Validation, Writing – original draft. MZ: Investigation, Validation, Writing – original draft. JT: Investigation, Methodology, Writing – review & editing. LM: Investigation, Writing – review & editing. HT: Investigation, Writing – review & editing. GH: Validation, Writing – review & editing. XT: Visualization, Writing – review & editing. LG: Validation, Writing – review & editing. XK: Methodology, Visualization, Writing – review & editing. KT: Conceptualization, Funding acquisition, Project administration, Supervision, Writing – review & editing. XB: Conceptualization, Methodology, Project administration, Supervision, Visualization, Writing – review & editing.
