# Supplementary material for: Inhibition of CX3CL1 by treadmill training prevents osteoclast-induced fibrocartilage complex resorption during TBI healing
Source: Front Immunol. 2024 Jan 12;14:1295163. doi: 10.3389/fimmu.2023.1295163 (PMC10811130; doi:10.3389/fimmu.2023.1295163)
Supplement: Supplementary file 1 [file DataSheet_1.pdf]

Table S1 Micro-CT raw data of tendon-bone insertion healing			
	N(n=6)	C(n=6)	T(n=6)
BV/TV(%)	6.578±0.490**	5.385±0.457	7.847±1.021****
BMD(g/cm3)	0.043±0.001****	0.021±0.004	0.051±0.011****
Data shown as mean±SD unless otherwise indicated. N,Normal group; C, Control group;T, Treadmill group.			
Statistical difference compared with C. Statistically significant (***P* < *0.05 and* ****$P < 0.01$).			

Table S2 Scores for each item in the histomorphometric scoring system for each sample									
Group	Fibrocartilage cell number	Fibrocartilage cell alignment	Collagen fibre continuity	Collagen fibre orientation	Tidemark	Cellularity	Vascularity	Inflammation	Total scores
C1	1	2	1	1	2	0	2	1	10
C2	0	2	2	2	1	1	1	1	10
C3	1	1	1	2	1	2	1	2	11
C4	1	2	1	1	0	1	2	1	9
C5	2	1	1	2	2	0	1	0	9
C6	2	2	0	1	2	1	1	1	10
T1	2	2	1	2	0	1	2	2	12
T2	1	2	2	2	1	2	1	1	12
T3	2	2	2	3	1	0	2	1	13
T4	2	2	2	1	0	2	1	2	12
T5	1	2	2	2	1	2	2	1	13
T6	2	1	2	2	2	1	3	1	14

Table S3 Target gene sequence list			
Gene	NCBI Gene ID	Primer	Sequence(5'-3')
RANKL	21943	Forward<br>Reverse	GCTCCGAGCTGGTGAAGAAA<br>CCCCAAAGTACGTCGCATCT
CTSK	13038	Forward<br>Reverse	CCAGTGTGGTTCCTGTTGG<br>TTGCCGTGGCGTTATACAT
NFATc1	18018	Forward<br>Reverse	CTCGAAAGACAGCACTGGAGCAT<br>CGGCTGCCTTCCGTCTCATAG
TRAP	11433	Forward<br>Reverse	CGTCTCTGCACAGATTGCAT<br>GAGTTGCCACACAGCATCAC
CX3CL1	20312	Forward<br>Reverse	GACCCCTAAGGCTGAGGAAC<br>AGAAGAGGAGGCCAAGGAAG
CX3CR1	13051	Forward<br>Reverse	GACGGTTGCATTTAGCCATT<br>TGCTCAGAACACTTCCATGC
GAPDH	14433	Forward<br>Reverse	TGACCACAGTCCATGCCATCACTG<br>CAGGAGACAACCTGGTCCTCAGTG

Table S4 Scores for each item in the histomorphometric scoring system for each sample									
Group	Fibrocartilage cell number	Fibrocartilage cell alignment	Collagen fibre continuity	Collagen fibre orientation	Tidemark	Cellularity	Vascularity	Inflammation	Total scores
C1	2	1	1	0	2	1	2	1	10
C2	1	1	2	2	1	0	1	1	9
C3	1	2	1	1	2	1	1	1	10
C4	1	1	1	2	1	0	2	2	10
C5	2	1	0	1	1	1	2	1	9
C6	2	2	2	1	1	0	1	1	10
AZD8797-1	1	2	2	1	1	2	3	2	14
AZD8797-2	2	2	1	2	1	2	2	2	14
AZD8797-3	2	1	2	2	1	1	1	2	12
AZD8797-4	1	2	2	1	1	1	2	2	12
AZD8797-5	1	3	2	1	2	1	1	2	13
AZD8797-6	2	2	1	2	1	2	2	1	13

Table S5 Histomorphometric scoring system for tendon-bone insertion healing					
		Score criteria			
Items		0	1	2	3
	Fibrocartilage cell number[a]	0%~25%	25%~50%	50%~75%	≥75.0%
	Fibrocartilage cell alignment	None	Unorganized	Moderate alignment	Highly aligned
	Collagen fibre	0%~25%	25%~50%	50%~75%	75%~100%
Tendon-to-bone interface	continuity[a]				
	Collagen fibre	0%~25%	25%~50%	50%~75%	75%~100%
	orientation[a]				
	Tidemark[a]	0%~25%	25%~50%	50%~75%	75%~100%
Cellularity[a]		>400%	300~400	200~300	<200
Vascularity[b]		>15	10~15	6~10	≤5
Inflammation		Abundant inflammatory cells	Moderate inflammatory cells	Minimal inflammatory cells	No inflammatory cells
Total scores		0~24			
a:The percentage is the relative value compared with the uninjured tendon bone interface tissue.					
b:Number of blood vessels per low power field (×10) from each sectionlow-power field.					

Table S6 Comparison and Screening Results between Groups

Name	Ratio_ Ms-TBI T_vs_Ms-TBI C	Ms-TBI T	Ms-TBI C
FLRG / Follistatin	2.00	24.5	12.3
Fractalkine	0.49	11.0	22.4
Frizzled-7	2.02	34.4	17.1
ICAM-1	2.30	25.8	11.2
IL-17R	2.15	34.4	16.0
IL-20 R alpha	2.61	20.9	8.0
L-Selectin	2.07	37.4	18.1
MIP-3 alpha	2.09	12.3	5.9
MIP-3 beta	2.96	11.0	3.7
MMP-9	0.22	4.3	19.2
PF-4	14.11	14.1	1.0
P-Selectin	2.53	6.8	2.7
Resistin	0.29	3.1	10.7
Serum Amyloid A1	4.61	17.2	3.7
Thrombospondin	3.23	81.0	25.0
WIF-1	2.07	5.5	2.7
WISP-1 / CCN4	0.46	8.6	18.6
